# MSCV-based retroviral plasmids expressing 3xFLAG-Sp-dCas9 for enChIP analysis

**DOI:** 10.1093/biomethods/bpab013

**Published:** 2021-07-09

**Authors:** Miyuki Yuno, Shoko Nagata, Toshitsugu Fujita, Hodaka Fujii

**Affiliations:** 1 Chromatin Biochemistry Research Group, Combined Program on Microbiology and Immunology, Research Institute for Microbial Diseases, Osaka University, 3-1 Yamadaoka, Suita, Osaka 565-0871, Japan; 2 Department of Biochemistry and Genome Biology, Hirosaki University Graduate School of Medicine, 5 Zaifu-cho, Hirosaki, Aomori 036-8562, Japan; 3 Research Division, Kobe Research Center, Epigeneron, Inc., CoLaborator Kobe 602, Kobe International Business Center, 5-5-2 Minatojima Minamimachi, Chuo-ku, Kobe 650-0047, Japan

**Keywords:** enChIP, dCas9, ChIP, chromatin immunoprecipitation, CRISPR, MSCV

## Abstract

Engineered DNA-binding molecule-mediated chromatin immunoprecipitation (enChIP) is a technology for purifying specific genomic regions to facilitate identification of their associated molecules, including proteins, RNAs, and other genomic regions. In enChIP, the target genomic region is tagged with engineered DNA-binding molecules, for example, a variant of the clustered regularly interspaced short palindromic repeats (CRISPR) system consisting of a guide RNA (gRNA) and a catalytically inactive form of Cas9 (dCas9). In this study, to increase the flexibility of enChIP and expand the range of target cells, we generated murine stem cell virus (MSCV)-based retroviral plasmids for expressing dCas9. We constructed MSCV-based retroviral plasmids expressing *Streptococcus pyogenes* dCas9 fused to a 3xFLAG-tag (3xFLAG-Sp-dCas9) and various drug resistance genes. We showed that by using these plasmids, it is feasible to purify target genomic regions with yields comparable to those reported using other systems. These systems might give enChIP users greater flexibility in choosing optimal systems for drug selection of transduced cells.

## Introduction

To understand the molecular mechanisms underlying regulation of genome functions such as epigenetic regulation and transcription, it is necessary to identify the regulatory molecules that bind to a genomic region of interest. We recently developed engineered DNA-binding molecule-mediated chromatin immunoprecipitation (enChIP) technology for the isolation of genomic regions of interest to facilitate identification of their associated molecules [1]. The engineered DNA-binding molecules that can be used to tag a target locus include transcription activator-like (TAL) proteins [2] and the clustered regularly interspaced short palindromic repeats (CRISPR) system [3–5] consisting of a guide RNA (gRNA) and a catalytically inactive form of Cas9 (dCas9) (see our recent review [6] for a comprehensive list of publications using CRISPR-based systems). Locus-tagging can be achieved by expression of engineered DNA-binding molecules in the cells to be analyzed (in-cell enChIP) [1]. Alternatively, it can be achieved *in vitro* using recombinant or synthetic engineered DNA-binding molecules (*in vitro* enChIP) [7]. After tagging with an engineered DNA-binding molecule, the locus is isolated by affinity purification and its associated proteins are identified by mass spectrometry (MS) [1, 8]. Associated nucleic acids such as RNAs [9, 10] and other genomic regions [11, 12] are identified by next-generation sequencing (NGS).

In this study, we generated murine stem cell virus (MSCV)-based retroviral plasmids expressing 3xFLAG-Sp-dCas9 and various drug resistance genes. Using these plasmids, we were able to purify target genomic regions with yields comparable to those reported using other systems. These systems might give enChIP users greater flexibility in choosing optimal systems for drug selection of transduced cells. In addition, they could be used to analyze different types of target cells.

## Materials and methods

### Plasmids

To construct MSCV expression plasmids expressing 3xFLAG-Sp-dCas9, the pMSCVneo, pMSCVhyg, and pMSCVpuro vectors (631461, Takara Bio, Kusatsu, Japan) were digested with *Bgl*II (1021A, Takara Bio) and *Xho* I (1094A, Takara Bio). After a blunting reaction, the plasmids were treated with bacterial alkaline phosphatase (*Escherichiacoli* C75) (2120A, Takara Bio). The cleaved vectors were purified by agarose gel electrophoresis and ligated with the coding sequence of 3xFLAG-Sp-dCas9, which was isolated from 3xFLAG-dCas9/pMXs-puro (Addgene, Watertown, MA, USA, #51240) [13] by digestion with *Pac* I (R0547, New England Biolabs, Ipswich, MA, USA) and *Not* I (1166A, Takara Bio).

To construct gRNA-hIRF-1 #12/pSIR-human CD2 (hCD2) (Addgene #135392), pSIR-hCD2 (Addgene #51143) [13] was digested with *Eco*R I (1040A, Takara Bio) and treated with bacterial alkaline phosphatase. The cleaved vector was purified by agarose gel electrophoresis and ligated with the gBlock targeting the *interferon* (*IFN*) *regulatory factor* (*IRF*)*-1* gene promoter, which was isolated from gRNA-hIRF-1 #12 (Addgene #61079) [1] by digestion with *Eco*R I.

A retroviral vector expressing a gRNA targeting the promoter region of the mouse c-*myc* gene, m-c-myc gRNA #1/pSIR-hCD2, was previously described [14].

The Addgene plasmid # of the newly made constructs are as follows: 3xFLAG-Sp-dCas9/pMSCVneo: 134982; 3xFLAG-Sp-dCas9/pMSCVhyg: 134323; 3xFLAG-Sp-dCas9/pMSCVpuro: 134983 and gRNA-hIRF-1 #12/pSIR-hCD2: 135392.

### Cell lines

The 293 T cell line was derived by transformation of human embryonic kidney (HEK) 293 cells with the SV40 large T antigen [15]. HT1080 is a human fibrosarcoma cell line purchased from ATCC (Manassas, VA, USA) (CCL-121) [16]. All the cells were cultured in Dulbecco’s modified Eagle’s medium (DMEM) (FUJIFILM Wako Pure Chemical, Osaka, Japan) supplemented with 10% fetal calf serum (FCS).

### Transduction and transfection of retroviral plasmids

For transduction of retroviral plasmids expressing 3xFLAG-Sp-dCas9 into HT1080 cells, 5.5 µg of each plasmid was transfected into 1 × 10^6^ of 293 T cells along with 5.5 µg of pPAM3 [17] using Lipofectamine 3000 (Thermo Fisher Scientific, Waltham, MA, USA). Two days after transfection, viral supernatant was harvested and used for infection of HT1080 cells. Infected cells were selected in culture media containing G418 (Nacalai Tesque, Kyoto, Japan) (0.8 mg/mL), hygromycin (Nacalai Tesque) (75 µg/mL) or puromycin (Nacalai Tesque) (0.75 µg/mL).

For transduction of retroviral plasmids expressing the gRNA into HT1080-derived cells expressing 3xFLAG-dCas9, 5.5 µg of gRNA-hIRF-1 #12/pSIR-hCD2 was transfected into 1 × 10^6^ of 293 T cells along with 5.5 µg of pPAM3 using Lipofectamine 3000. Two days after transfection, viral supernatant was harvested and used for infection of HT1080 cells expressing 3xFLAG-dCas9. Cells expressing 3xFLAG-Sp-dCas9 and gRNA were sorted by MACS as described below.

For transduction of retroviral plasmids expressing 3xFLAG-Sp-dCas9 into NIH 3T3 cells, 5.5 µg of each plasmid was transfected into 1 × 10^6^ of Plat-E cells [18] using Lipofectamine 3000. Two days after transfection, viral supernatant was harvested and used for infection of NIH 3T3 cells. Infected cells were selected in culture media containing G418 (0.7 mg/mL), hygromycin (100 µg/mL) or puromycin (1.5 µg/mL).

For expression of gRNA targeting the c-*myc* promoter into NIH 3T3-derived cells expressing 3xFLAG-Sp-dCas9, 4 × 10^5^ cells expressing 3xFLAG-Sp-dCas9 were plated in a well of six-well plate. Next day, 4 µg of m-c-myc gRNA #1/pSIR-hCD2 was transfected using Lipofectamine 3000. Three days after transfection, cells were harvested for enChIP analysis.

### Immunoblot analysis

Cytoplasmic extracts and nuclear extracts were prepared using the NE-PER Nuclear and Cytoplasmic Extraction Reagents (Thermo Fisher Scientific). Ten micrograms of cytoplasmic extract from HT1080-derived cells and 8 μg of nuclear extracts from NIH 3T3-derived cells were subjected to immunoblot analysis with anti-FLAG M2 antibody (Ab) (F1804, Sigma–Aldrich, Saint Louis, MO, USA) as described previously [1].

### Magnetic-activated cell sorting (MACS) sorting and flow cytometry

MACS sorting of hCD2 (+) cells was performed using CD2 Microbeads (130-091-114, Miltenyi Biotec B.V. & Co. KG, Bergisch Gladbach, Germany). To monitor the results of MACS sorting, cells were stained with phycoerythrin (PE)-conjugated anti-hCD2 Ab (347597, BD Life Sciences, San Jose, CA, USA) for 30 min at 4°C. Flow cytometry was performed on a FACSCalibur (BD Life Sciences), and data were analyzed using the FlowJo software (BD Life Sciences).

### enChIP real-time PCR

enChIP-real-time PCR was performed as previously described [13]. The primers used in this study were reported previously [1, 14].

## Results and discussion

### MSCV retroviral expression systems for enChIP

**FIGURE 1:**
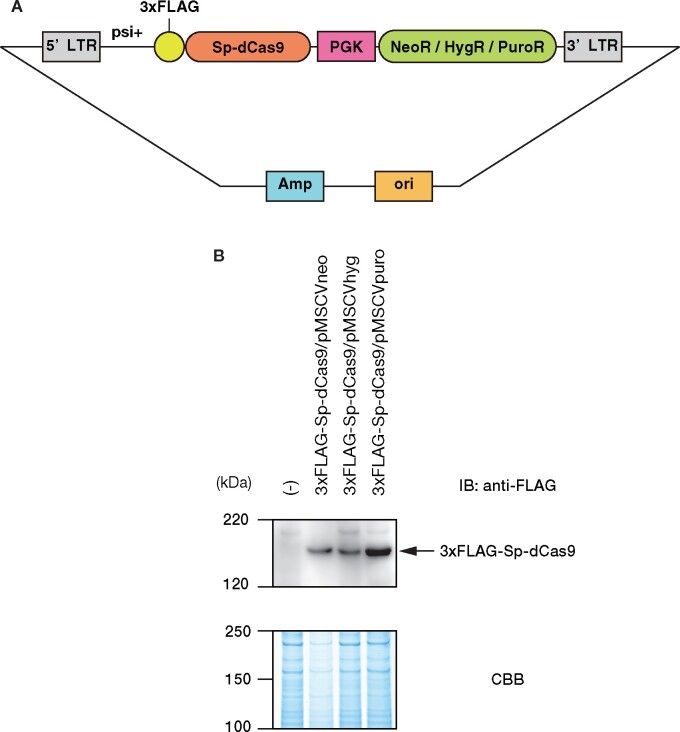
enChIP systems using MSCV–based retroviral expression vectors. (a) Schematics of MSCV–based retroviral expression constructs of 3xFLAG–Sp–dCas9. (b) Expression of 3xFLAG-Sp-dCas9. Immunoblot analysis was performed with anti-FLAG Ab. Coomassie Brilliant Blue (CBB) staining is shown as a protein loading control.

To increase the flexibility of enChIP and expand the range of target cells, we constructed retroviral plasmids expressing *S. pyogenes* dCas9 fused with the 3xFLAG-tag (3xFLAG-Sp-dCas9), using the MSCV system along with various drug selection markers (Fig. 1A).

**FIGURE 2:**
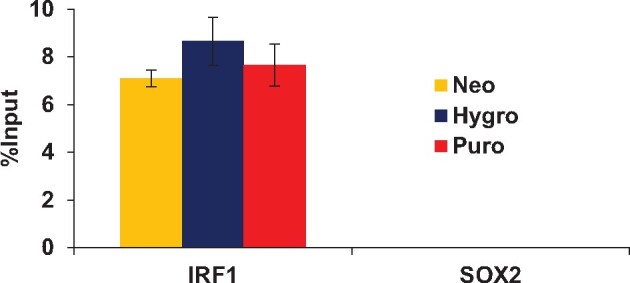
Isolation of the *IRF–1* locus by enChIP systems using MSCV–based retroviral expression vectors. Real–time PCR analysis was performed on chromatin complexes isolated by enChIP. An irrelevant locus (*SOX2*) was analyzed as a negative control. Error bars represent the standard deviations of technical replicates (n = 4).

To confirm the performance of the system, we transduced the retroviral plasmids into HT1080, a human fibrosarcoma cell line. After drug selection, expression of 3xFLAG-Sp-dCas9 was confirmed by immunoblot analysis with Ab against the FLAG-tag (Fig. 1B). Subsequently, gRNA-hIRF-1 #12/pSIR-hCD2, a self-inactivating retroviral vector expressing a sgRNA targeting human *IRF-1* gene promoter and the hCD2 selection marker protein, was transduced into the HT1080-derived cells expressing 3xFLAG-Sp-dCas9. Two days after transduction, cells expressing the sgRNA (hCD2 (+) cells) were selected by MACS sorting. After expansion, 2 × 10^6^ cells were subjected to enChIP analysis. Briefly, the cells were crosslinked with formaldehyde and the crosslinked chromatin was fragmented by sonication. Subsequently, fragmented chromatin tagged with the CRISPR complex was purified using anti-FLAG Ab. As shown in Fig. 2, the yields of enChIP were comparable to those reported using other systems [13].

**FIGURE 3:**
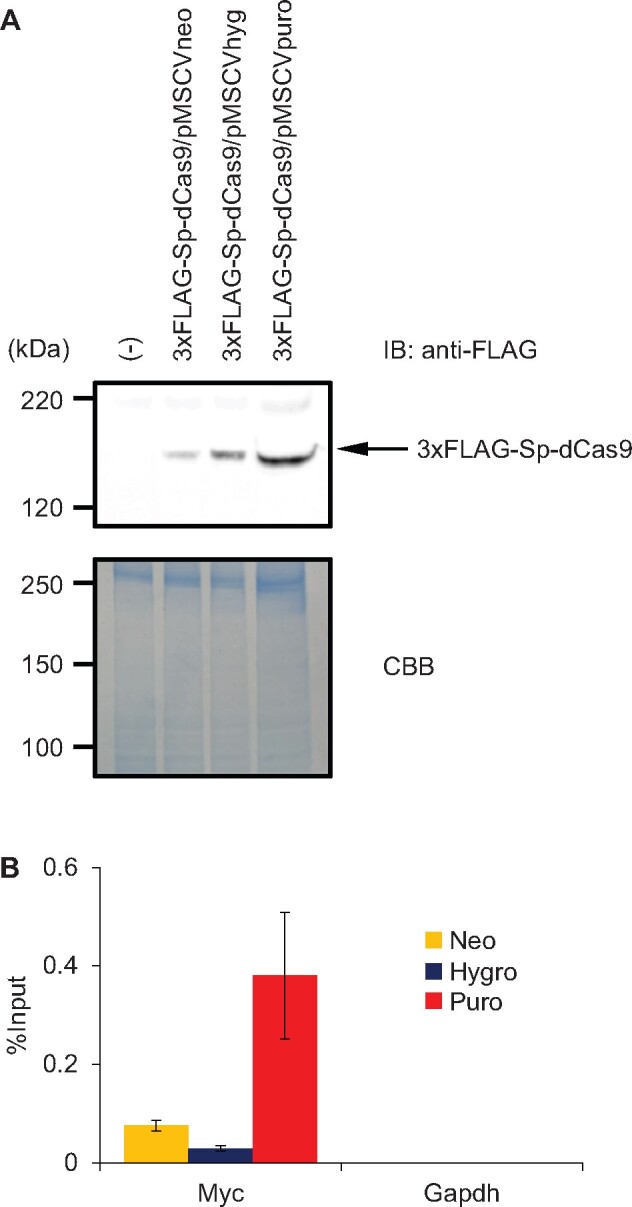
Isolation of the mouse c-*myc* locus by enChIP systems using MSCV-based retroviral expression vectors. (**A**) Expression of 3xFLAG-Sp-dCas9. Immunoblot analysis was performed with anti-FLAG Ab. Coomassie Brilliant Blue (CBB) staining is shown as a protein loading control. (**B**) Isolation of the mouse c-*myc* locus by enChIP systems using MSCV-based retroviral expression vectors. Real-time PCR analysis was performed on chromatin complexes isolated by enChIP. An irrelevant locus (*Gapdh*) was analyzed as a negative control. Error bars represent the range of duplicate experiments.

We next examined if the MSCV retroviral expression system works in other cell lines and target loci. We transduced the retroviral plasmids into a mouse fibroblast cell line, NIH 3T3. After drug selection, expression of 3xFLAG-Sp-dCas9 was confirmed by immunoblot analysis with Ab against the FLAG-tag (Fig. 3A). Subsequently, m-c-myc gRNA #1/pSIR-hCD2, an expression vector expressing an sgRNA targeting mouse c-*myc* gene promoter, was transfected into the NIH 3T3-derived cells expressing 3xFLAG-Sp-dCas9. Three days after transfection, cells were subjected to enChIP analysis. As shown in Fig. 3B, the c-*myc* locus was specifically enriched by enChIP.

These results suggested that the enChIP system using the MSCV retroviral vectors can be used for purification of target genomic regions and subsequent downstream applications such as identification of molecules associated with the target genomic regions.

## Conclusions

In this study, we developed enChIP systems using MSCV-based retroviral expression vectors with various selection markers. To the best of our knowledge, there is currently no retroviral plasmid expressing 3xFLAG-Sp-dCas9 and the hygromycin resistance gene in Addgene. These systems might give enChIP users greater flexibility in choosing optimal systems for drug selection of transduced cells. In addition, they could be used to analyze target cells that might be difficult to analyze using other systems.

## Limitations

Although the tropism of MSCV-based retroviral vectors has been extensively analyzed, only two cell lines (the HT1080 and NIH 3T3 cell lines) were analyzed in this study. In addition, although it has been shown that many different loci can be analyzed using 3xFLAG-Sp-dCas9, only two loci (the *IRF-1* promoter and c-*myc* promoter) were targeted in this study. Further studies will be necessary to determine which cell types and which loci can be analyzed using this system. Further studies might also be necessary to assess the utility of these systems in combination with MS and NGS to identify molecules associated with target genomic regions.

In this study, we used hCD2 as a marker to select gRNA (+) cells. However, this might not work for cells expressing endogenous hCD2, such as acute lymphocytic leukaemia (ALL) cell lines. Therefore, other selection markers should be used for these cells. In this regard, we have already developed and made available through Addgene other selection markers including drug resistance genes and fluorescent proteins such as green fluorescent protein and DsRed-Express2 red fluorescent protein [13]. One of the advantages of hCD2 and other cell surface markers for selection is that cells expressing these markers can be purified directly using magnetic-based cell isolation systems and without the need for expensive instruments such as flowcytometric cell sorters.
